# Land-Use and Land-Cover Classification in Semi-Arid Areas from Medium-Resolution Remote-Sensing Imagery: A Deep Learning Approach

**DOI:** 10.3390/s22228750

**Published:** 2022-11-12

**Authors:** Kamran Ali, Brian A. Johnson

**Affiliations:** 1 Institute of Geographical Information Systems, School of Civil and Environmental Engineering, National University of Sciences and Technology (NUST), Islamabad 44000, Pakistan; 2 Natural Resources and Ecosystem Services Area, Institute for Global Environmental Strategies, Hayama 240-0115, Kanagawa, Japan

**Keywords:** CNN, LULC classification, semi-arid regions, Sentinel-2

## Abstract

Detailed Land-Use and Land-Cover (LULC) information is of pivotal importance in, e.g., urban/rural planning, disaster management, and climate change adaptation. Recently, Deep Learning (DL) has emerged as a paradigm shift for LULC classification. To date, little research has focused on using DL methods for LULC mapping in semi-arid regions, and none that we are aware of have compared the use of different Sentinel-2 image band combinations for mapping LULC in semi-arid landscapes with deep Convolutional Neural Network (CNN) models. Sentinel-2 multispectral image bands have varying spatial resolutions, and there is often high spectral similarity of different LULC features in semi-arid regions; therefore, selection of suitable Sentinel-2 bands could be an important factor for LULC mapping in these areas. Our study contributes to the remote sensing literature by testing different Sentinel-2 bands, as well as the transferability of well-optimized CNNs, for semi-arid LULC classification in semi-arid regions. We first trained a CNN model in one semi-arid study site (Gujranwala city, Gujranwala Saddar and Wazirabadtownships, Pakistan), and then applied the pre-trained model to map LULC in two additional semi-arid study sites (Lahore and Faisalabad city, Pakistan). Two different composite images were compared: (i) a four-band composite with 10 m spatial resolution image bands (Near-Infrared (NIR), green, blue, and red bands), and (ii) a ten-band composite made by adding two Short Wave Infrared (SWIR) bands and four vegetation red-edge bands to the four-band composite. Experimental results corroborate the validity of the proposed CNN architecture. Notably, the four-band CNN model has shown robustness in semi-arid regions, where spatially and spectrally confusing land-covers are present.

## 1. Introduction

Detailed LULC information over large areas is essential for a wide range of urban and natural resource management issues, including urban area mapping [1,2], monitoring urban expansion, and infrastructure planning [3]. Remote-sensing images are the most important data sources for accurate LULC information [4,5], which provide efficient information about the Earth’s surface at a low cost [6]. Remote-sensing satellites range from coarse spatial resolution (MODIS and AVHRR, etc.) to medium-resolution (Sentinel-2 and Landsat-8, etc.) and high-resolution (GeoEye, Ikonos, QuickBird, Gaofen, etc.) satellites [7,8,9,10]. However, it is often expensive to acquire high-resolution remote-sensing satellite imagery for large areas (most is commercial imagery) [11,12], and there may not be recent cloud-free imagery available for a site of interest due to the infrequent acquisition of high-resolution imagery (compared to medium-resolution imagery).

Medium-resolution remote-sensing imagery is perhaps the most important data source for generating maps of LULC over large areas due to its ability to provide near-global coverage of the Earth’s surface at a high frequency, e.g., every 5 or 8 days for Sentinel (-2a/2b) and Landsat (-8/-9) satellite data, respectively [12,13]. LULC classification in arid/semi-arid regions is a challenging task due to high interclass spectral similarity of urban and non-urban (e.g., bare land or fallow cropland) LULC features [1,14].

Freely available medium-resolution remote-sensing imagery (e.g., 10–60 m resolution Sentinel-2 data) is a valuable data source for LULC classification of semi-arid regions due to its low cost and high temporal resolution. There is a lack of studies on the utilization of medium-resolution remote-sensing data for mapping LULC in semi-arid regions (i.e., spatially and spectrally similar LULC features), particularly over large geographic areas, e.g., at city, provincial, and national scales. Thus, accurate LULC classification over large semi-arid areas is still a challenge for the remote-sensing community.

The remote-sensing community has developed and applied many different classification methods for LULC mapping, ranging from conventional methods based on image statistics—e.g., Bayesian, Maximum Likelihood, or ISO Clustering methods [15]—to advanced Machine Learning (ML) methods, such as Support Vector Machine (SVM), Light Gradient Boosting Machine (LGBM) [5,16], Random Forest (RF), single Decision Trees (DTs), and K-nearest neighbors (KNN) [6,17,18]. More recently, DL methods—e.g., CNN [8,19,20,21]—have also been applied for LULC mapping in a variety of different types of landscapes. CNNs have also been used for various other applications in built-up areas, including analysis of concrete (crack detection and torsional capacity) [22]. Notably, these DL-based LULC classification methods have often outperformed the conventional and ML classification methods when there is a sufficient amount of training data available (while ML approaches may give similar or superior performance when the training data is very limited) [23]. A major advantage of DL approaches is their ability to automatically learn the most useful spectral and contextual features from the training set to help distinguish between the spectrally similar LULC classes [24,25]. With ML approaches, this can often be a complex and time-consuming process requiring the use of feature-selection algorithms and/or expert knowledge/trial-and-error [26].

Unfortunately, there are few studies on the utilization of medium-resolution remote-sensing data for mapping of large semi-arid regions using DL models. As one example, [27] introduced a new type of Deep CNN (DCNN) for LULC classification using medium-resolution remote-sensing imagery (Landsat-8). In this study, seven different LULC classes comprising cultivated land, forest, grassland, wetland, water, construction land and bare land were considered. The DCNN model achieved an overall accuracy of 82%, and improved accuracy by 5% and 14% as compared to SVM and MLC classifiers, respectively. In [28], researchers designed a patch-based CNN system for the classification of Landsat satellite imagery into eight LULC classes (water, agriculture, forested wetland, barren land, trees, high- and low-intensity urban, and non-forested wetland), and found that the patch-based CNN achieved an overall accuracy of 89.26%, outperforming a pixel-based CNN, a pixel-based NN, and a patch-based NN classifier by a margin of 24.23%, 24.36%, and 11.52%, respectively. The proposed patch-based architecture is complex; additionally, a large number of training and testing samples are required to reduce overfitting and underfitting (the proposed model is trained on 150,000 iterations, which requires much computational power and time).

In Song et al. [9], researchers introduced a Lightweight CNN (LCNN) for the LULC classification of medium-resolution remote-sensing imagery (Landsat-8) and found that the LCNN had significantly higher accuracy than the pixel-based SVM, RF, and KNN algorithms. The proposed LCNN model attained a high classification accuracy with fewer training samples and lower computational power than the traditional CNNs. In Hervadi and Miranda [29], the authors conducted a study on LULC classification of Sentinel-2 imagery using CNN. In this study, different texture features (e.g., homogeneity, rectangular fit, shape, and brightness) and vegetation indices were used along with reflectance data for LULC classification. A comparative analysis of CNN was performed with a Gradient Boosting Machine (GBM) algorithm. CNN achieved 0.98 mean training and testing accuracy after using different texture features and vegetation indices along with Sentinel-2 data. However, it is a very time-consuming task to extract the texture features and vegetation indices.

While several DL-based methods have been proposed for LULC classification using medium-resolution remote-sensing images, many knowledge gaps remain. For example, there is still little information on the utility of combining/utilizing different spectral bands of medium-resolution satellite sensors for LULC mapping, or on mapping of LULC in arid/semi-arid regions using DL methods. To the best of our knowledge, there is no detailed study involving the use of DL methods to classify LULC in semi-arid regions using Sentinel-2 imagery. This is a significant knowledge gap due to the high potential of Sentinel-2 data for LULC mapping over wide geographic areas, as Sentinel-2 has 13 spectral bands with spatial resolutions ranging from 10–60 m and a revisit time of only 5 days. It would be interesting to further explore the potential of Sentinel-2 data to evaluate, e.g., which spectral band combinations yield better classification results. Therefore, the aim of this scientific research is as follows:

- To apply a 2D CNN architecture with fixed hyperparameters for LULC classification in semi-arid regions using medium-resolution remote-sensing imagery (Sentinel-2 data).
- To test the transferability of CNNs for semi-arid LULC classification in semi-arid regions.
- To evaluate and analyze the spectral bands, which can provide maximum class separability, minimize spectral confusion, and reduce the required computational power.

### Overview of DL CNNs

CNN is a class of feed-forward neural networks. Traditional ML algorithms need extracted features for classification, but the CNN automatically learns features. It automatically learns complex information from data and arranges it from low-level to high-level. It was originally designed to process data in the form of multiple arrays. Remote-sensing image data is in the form of multiple arranged arrays of pixels; this is why CNN is selected for remote-sensing image classification [30]. It extracts information from data in multiple stages. Each stage consists of, usually, three types of layers: (1) convolutional layers, (2) non-linear layers, and (3) pooling layers. These layers are connected to one or more fully connected layers [26]. The deeper CNN architecture has dropout and batch normalization layers [7].

The convolutional layers take three-dimensional (3D) input data (x × y × f), where ‘x’ and ‘y’ are the image patch dimensions and ‘f’ is the number of features. The convolutional filters extract high-level information from the training data by moving the window (kernel). The output is a 3D shape (x × y × z) composed of a ‘z’-feature map of size x × y. The pointwise nonlinear activation function layer is applied to each component in the convolutional layer, which computes the output features map. There are many activation functions, e.g., Rectified Linear Unit (ReLu), softmax, sigmoid, hyperbolic tangent (tanh) and maxout. The most commonly used activation functions for multi-class classification problems are ReLu and softmax [3,7,26,28].

The pooling layers reduce the spatial dimension of the convolutional layers’ output feature vector and extract the most useful high-level features with a moving kernel window [24,31,32]. There are multiple pooling layers, such as max pooling, average pooling, and mean pooling layers. The max pooling layer is the most commonly used layer in CNN. The pooling layer helps in reducing overfitting, as it extracts high-level features from the features map [33,34].

All the layers are connected to fully connected layers (dense layers). There is a flattening layer that converts the extracted features from the previous layers into a one-dimensional (1D) feature vector [35]. The dense layers take the 1D feature vector as an input. The dense layers consist of a number of neurons. Each neuron is composed of weight, bias, and an activation function. The last dense layer has an output layer that produces the classification result [34].

## 2. Materials and Methods

### 2.1. Study Areas

#### 2.1.1. Training Sites

**Figure 1:**
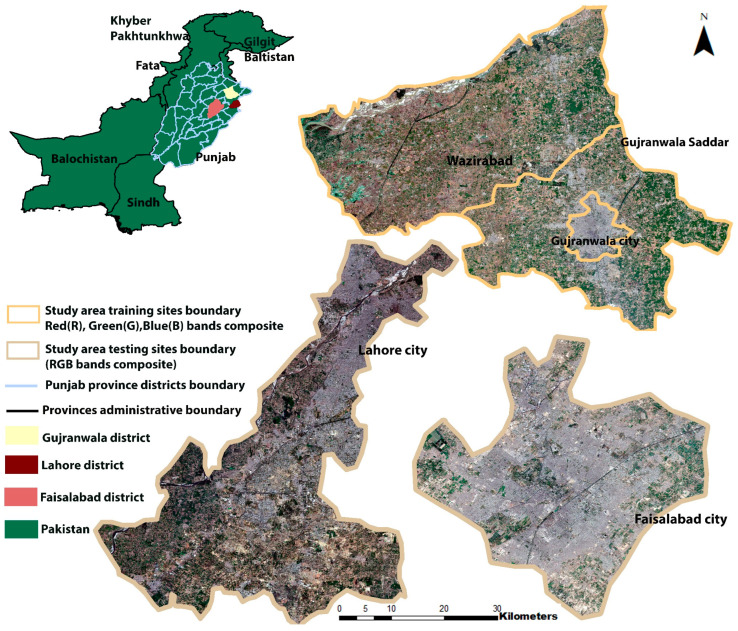
Study area map.

The study areas considered for this research consist of Gujranwala city and the surrounding peri-urban areas, including Gujranwala Saddar and Wazirabad tehsils (townships), which are located in the Gujranwala district of Punjab, Pakistan (Figure 1). The study area is situated in the heart of Rachna Doab, a strip of land between the Chenab River to the north and the Ravi River to the south. It is the 5th most populous region in Punjab, Pakistan. It has a semi-arid climate. It is characterized by high temperatures during summer (June to September), ranging between 36 °C and 42 °C (97 °F and 108 °F), and low temperatures during the winter season (November to February), ranging between 7 °C and 15 °C (45 °F and 59 °F). It receives the highest precipitation during the monsoon season (July and August). During other months, the average rainfall is about 25 mm (0.98 inches) [36]. It was specifically selected because of the diversity of LULC found in the area.

The area is composed of complex-structured (i.e., spatial and spectrally similar) LULC features, namely, barren land, settlements (built-up/urban land), vegetation, water bodies (rivers, canals, streams, etc.), and fallow land. There is unplanned LULC change occurring in this study area (e.g., growth of settlements) which poses a challenge for LULC monitoring of the site, particularly for the spectrally confused LULC features such as settlement, barren land, and fallow land.

#### 2.1.2. Testing Sites

Two additional cities in Pakistan, Lahore and Faisalabad, were considered for testing the DL models. These cities have a semi-arid climate. Lahore city is located in the Lahore district of the province of Punjab. The Ravi River flows at the north of the city of Lahore. It is the most populous city in the Punjab province. During summer, the highest temperature varies between 39 °C and 47 °C (102 °F and 117 °F), whereas December, January, and February are the coldest months [37]. The city of Faisalabad is located in the Faisalabad district of Punjab. Faisalabad is the second most populous district in the Punjab province. It has a very hot climate. During the summer, the maximum temperature reaches up to 50 °C (122 °F); mean maximum and minimum temperatures vary between 39 °C and 27 °C (102 °F and 87 °F). During the winter (December, January, and February), the temperature lies between 21 °C and 6 °C (70 °F and 43 °F) [38].

These are metropolitan cities, and have a diversity of unplanned LULC features. These cities have a less planned LULC structure than the study area sites described in Section 2.1.1. Therefore, these were deemed as suitable sites to test the performance of the trained DL models on unseen semi-arid regions.

### 2.2. Methodology

**Figure 2:**
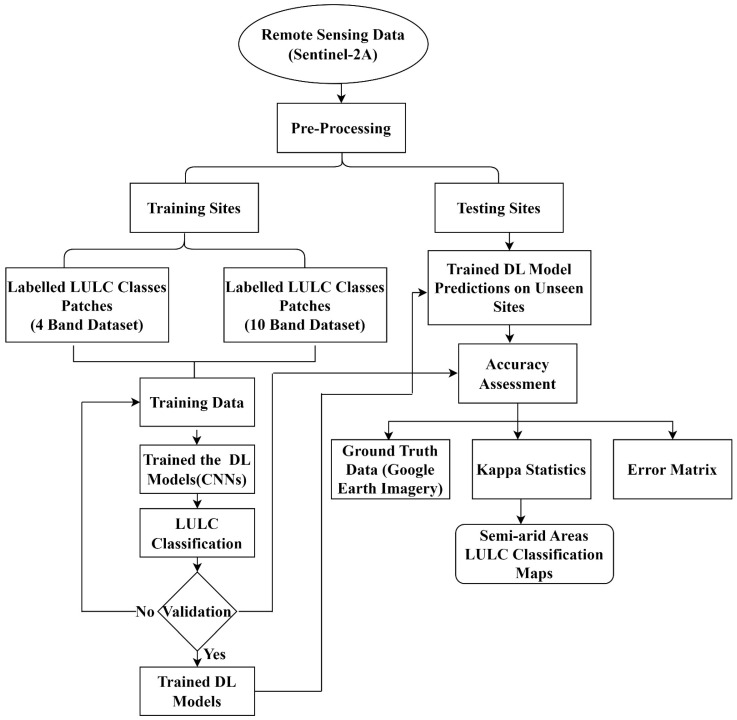
The methodology flowchart for semi-arid region classification using CNNs.

Figure 2 illustrates a detailed workflow of the methodology used in this study. The first step comprised the acquisition of Sentinel-2 imagery. In the second step, datasets were pre-processed and LULC classification was performed using CNN. In the last step, accuracy assessment was performed for the evaluation of results.

#### 2.2.1. Satellite Data Acquisition

This study used medium-resolution Sentinel-2 data. Sentinel-2′s Multi-Spectral Instrument (MSI) consists of 13 spectral bands, of which 4 bands have a 10 m spatial resolution, 6 bands have a 20 m spatial resolution, and 3 bands have a 60 m spatial resolution. Sentinel-2 is the first freely available satellite data that provides 4 spectral bands (blue, green, red, and NIR) at 10 m resolution (Landsat 7/8/9 have only 1 panchromatic band with a 10 m spatial resolution). Sentinel-2 level-2A product data contains Bottom of Atmospheric Reflectance (BOA) values, and the images are geometrically, radiometrically, and atmospherically corrected. For this study, four cloud-free Sentinel-2 tiles were downloaded from Copernicus Open Access Hub (https://scihub.copernicus.eu/ (accessed on 27 August 2021)). The acquisition date for the study area images was 14 October 2020 and 27 October 2020.

#### 2.2.2. Sentinel-2 Data Pre-processing

This study used ten spectral bands of Sentinel-2 data, including the blue, green, red, and NIR bands, as well as four vegetation red-edge bands and two SWIR bands, as described in Table 1. The remaining three bands (i.e., bands 1, 9, and 10) were not used because these bands are related to coastal and atmosphere-related applications, e.g., estimating water turbidity and cloud cover, and are not typically used for LULC mapping applications. Bands 5, 6, 7, 9, 11, and 12 were resampled from 20 m spatial resolution to 10 m spatial resolution using nearest-neighbor interpolation to integrate them with the 10 m spatial resolution bands. Nearest-neighbor interpolation is a resampling technique widely used in remote sensing to down-sample satellite image pixels [39]. Two different band composites were created for this study, as follows:

- A 4-band composite was created by using NIR, green, blue, and red bands.
- A 10-band composite was created by adding the two SWIR bands and four vegetation red-edge bands to the 4-band composite.

No extra indices or features were used for classification because they have limited contributions to LULC classification using CNN methods [40].

After the creation of band composites, the Region of Interest (ROI) was extracted from the stacked images using ROI boundary shapefiles.

#### 2.2.3. Dataset Preparation

Five different LULC classes—settlement, barren land, fallow land, vegetation, and water bodies—were considered for classification of the Sentinel-2 composite images (4-band and 10-band images). Patch-based CNN was used for image classification in this study, as it typically performs better than the pixel-based CNN method in terms of classification accuracy [9,27,28]. The CNN patch size is determined on the basis of the LULC features to be extracted from the satellite imagery, e.g., their size and spatial structure, considering the image spatial resolution [7]. In this study, a 5 × 5 pixel patch size was selected due to the complex structure of LULC features.

Next, training data was collected. In total, 2400 training patches of 5 × 5 pixel dimensions were extracted for each LULC class. These training patches were manually labeled through visual interpretation using high-resolution Google Earth imagery. Table 2 presents the details of training samples used for LULC classification.

#### 2.2.4. LULC Classification

LULC classification was performed by using CNN, with 4-band and 10-band composite datasets as inputs. The classification was performed in Google Colab Pro, and the CNN implemented in Google’s TensorFlow.

#### 2.2.5. The Proposed 2D CNN

**Figure 3:**
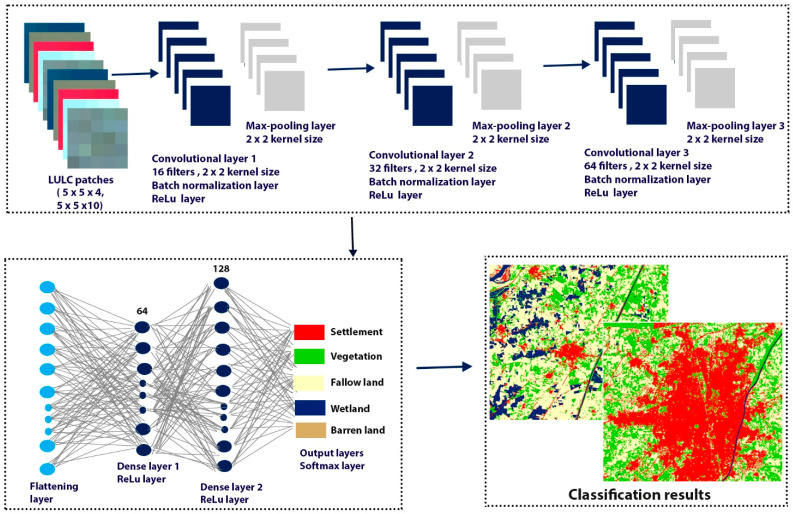
The architecture of the proposed CNN for semi-arid region LULC classification.

In this study, we proposed a CNN architecture that is appropriate for the LULC classification of semi-arid regions and that efficiently extracts features from the images. The CNN model was fed with the 3D input shape (size × size × number of bands) with a patch size of 5 × 5 × 4 and 5 × 5 × 10 pixels. The proposed architecture comprises three convolutional layers. The first, second, and third convolutional layers have a filter of 16, 32, and 64, respectively. All the convolutional layers have been used with kernel size (2,2) and stride (1,1). Two max pooling layers with kernel size (2,2) were used because max pooling layers reduce the spatial dimension and pick the maximum pixel value from the training images [24,31,32]. Two batch normalization layers were used to increase the training speed. Five dropout layers were used: three dropout layers with size (0.2) and two dropout layers with size (0.5) were used to avoid overfitting in the training process. One flattening layer was used to convert the 2D data vector into the 1D vector used as input to the dense layers. Two dense layers were used with the ReLu activation function. One dense layer was used with 64 neurons and the second dense layer was used with 128 neurons. The ReLu activation function performs the element-wise operations and sets all negative pixels to zero, outputting a rectified feature map. Finally, one output layer was used with the softmax activation function. Figure 3 shows the architecture of the proposed CNN for semi-arid region LULC classification.

##### Parameter Optimization

CNNs architecture requires setting several parameters, including the number of epochs, learning rate, batch size, activation function, optimizer, loss function, etc. Values of these parameters can have a significant impact on classification accuracy [41]. Thus, there is a need to fine-tune these parameter settings to get the desired more-accurate output (classified LULC map). In this study, we have performed experiments with several different batch sizes (32, 64, and 128) and numbers of epochs (30, 50, 100, 150, 200, 250, and 300). The Adam optimizer was used to reduce the training cost and computational power, and to have the dominant effect of achieving higher classification accuracy [28,42]. The model was also experimented with using different loss functions, such as categorical cross-entropy class, sparse categorical cross-entropy function, categorical cross-entropy functions, and sparse categorical cross-entropy class. The ReLu activation function was used as an activator in all the hidden layers because it allows the model to run fast and perform better [43]. The softmax activation function is used in the output layer. Multiple experiments were performed with different learning rate values such as 0.1, 0.01, 0.001, 0.0001, etc. The parameters and their values are described in Table 3.

#### 2.2.6. Performance Evaluation

The classification results were evaluated using qualitative and quantitative methods. For the qualitative assessment, classification maps were visually compared with the referenced data (Google Earth imagery). For the quantitative assessment, we randomly generated 850 points in each of the training and testing sites based on the classified LULC maps and used them for calculating Overall Accuracy (OA), User Accuracy (UA), Producer Accuracy (PA), and kappa coefficient based on visual interpretation of the Google Earth imagery and the LULC maps. OA is calculated by dividing the total number of correctly classified points (sum of the diagonal points) by the total number of ground truth points in the confusion matrices. The PA is calculated by dividing the correctly classified points by the total number of points (points in the column of each class) classified as the LULC class. The UA is calculated by dividing the total number of correctly classified points by the total number of ground truth points (points in the rows of each class) [20,44,45]. A kappa analysis yields a K statistic, which is a quantitative measure of agreement or accuracy of correctly classified points. K = 1 indicates the ideal agreement; K closer to 1 means that there is perfect agreement in the correctly classified points [46]. The K statistic was computed as:

(1) $$K=\frac{N\sum_{i,j=1}^{n}X_{ij}-\sum_{i,j=1}^{n}(Y_{i}\times Z_{j})}{N^{2}-\sum_{i,j=1}^{n}(Y_{i}\times Z_{j})}$$

where *N* is the total number of ground truth points, *n* is the total number of LULC classes, $X_{ij}$ is the sum of correctly classified points in row i and column j, $Y_{i}$ is the total number of points in rows, and $Z_{j}$ is the total number of points in the columns.

## 3. Results

### 3.1. Qualitative Analysis of Training Site Land Cover Maps

**Figure 4:**
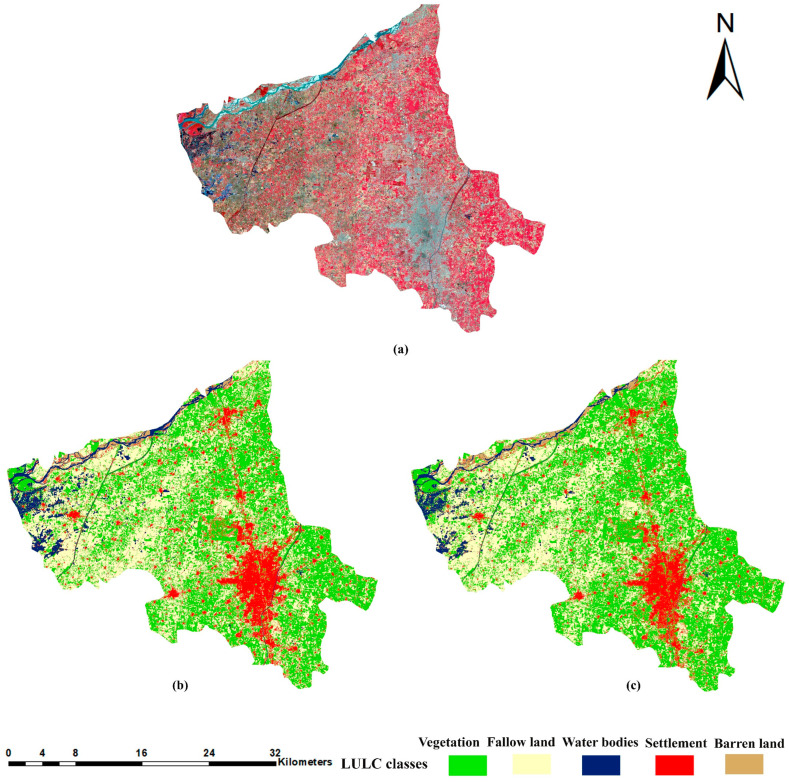
Comparison between the classification results of training site study area. (**a**) False-color composite image of training sites study area. (**b**) Classification result of 4-band CNN model. (**c**) Classification result of 10-band CNN model.

**Figure 5:**
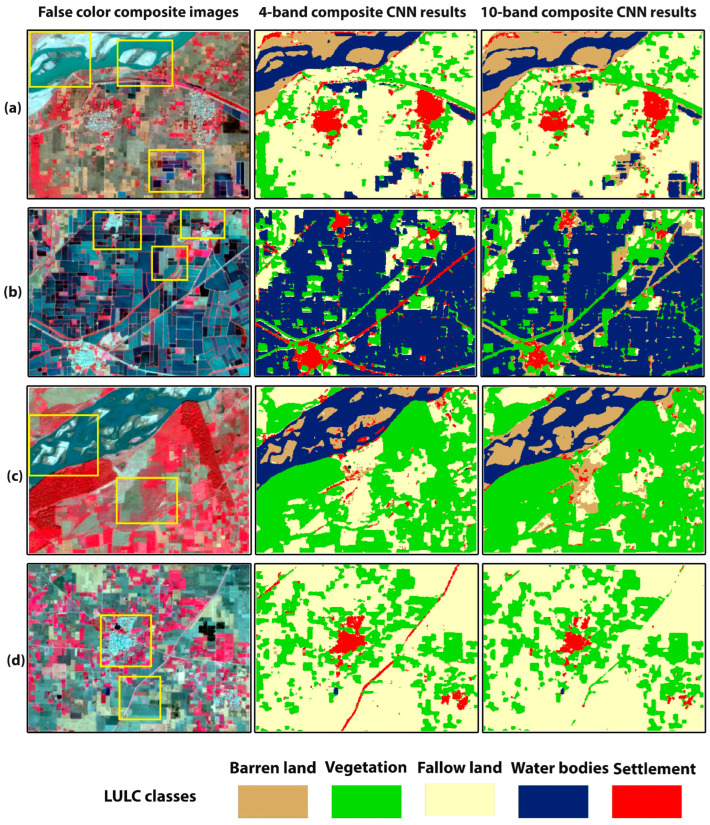
(**a**–**d**) are smaller subsets of training site classified map. First column shows the false-color composite images, second and third columns show the classification maps of 4- and 10-band CNN models, respectively. The yellow rectangles highlight areas with differences between the classification results of 4- and 10- band CNN models.

**Figure 6:**
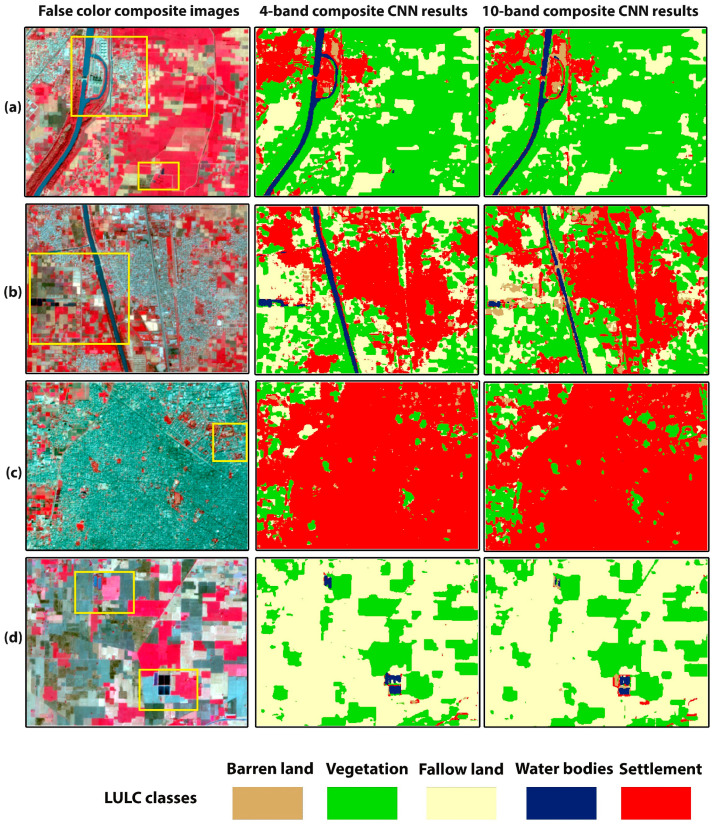
(**a**–**d**) are smaller subsets of training site classified map. First column shows the false-color composite images, second and third columns show the classification maps of 4- and 10-bands CNN models, respectively. The yellow rectangles highlight areas with differences between the classification results of 4- and 10- band CNN models.

Figure 4 shows the comparison between the 4- and 10-band CNN models’ classification results of the training site study area. Figure 5 and Figure 6 show smaller subsets of these maps in more detail. The false-color composite images were used as a reference in comparison with the classification maps. Figure 5a,c depicts an area which is mainly characterized by the river, fallow land, wet crops (irrigated crops), and small settlement region. It can be observed that, in the rectangle’s area, the 10-band CNN model could not classify the river pixels better than the 4-band CNN model. The 10-band CNN model misclassified the river area as barren land area and could not preserve the exact geometry of the landcover scene. In Figure 5c, 4-band CNN results show some pixels of barren land are misclassified as settlement, but there is no misclassification between these classes in the 10-band CNN results. Figure 5b represents the rural area characterized by wet crops. In the rectangle’s area, the 10-band CNN model confused settlement, fallow land, and water bodies classes. It misclassified the settlement and fallow land areas as barren land, while the 4-band CNN model classified these landcover areas well. Figure 5d involves the rural area with vegetation, fallow land, and a small settlement area. The 10-band CNN model misclassified some settlement areas as fallow land. The 4-band CNN model classified the roads, but the 10-band CNN model could not. Figure 6a represents the rural area with canal, fallow land, vegetation, and settlement areas. The 10-band CNN model misclassified the canal area as a settlement area, and wet crop as fallow land. On the other hand, the 4-band CNN model has correctly classified these landcover classes. Figure 6b,d depicts a canal area with some settlement and fallow land areas. The 10-band CNN model shows compactness in the canal area as compared to the 4-band CNN model results. The 10-band CNN model misclassified the wet crop area as barren land class. Figure 6c shows the urban area; in the rectangle’s area, it can be seen that the 10-band CNN model has misclassified some urban areas as fallow land and barren land class. The qualitative analysis of these landcover maps demonstrates that the 4-band CNN model results are better than the 10-band CNN model results. The spectral confusion between the highly spectrally confused LULC classes is higher in the 10-band CNN model as compared to the 4-band CNN model results.

### 3.2. Quantitative Analysis of Training Site Classification Results

**Figure A1:**
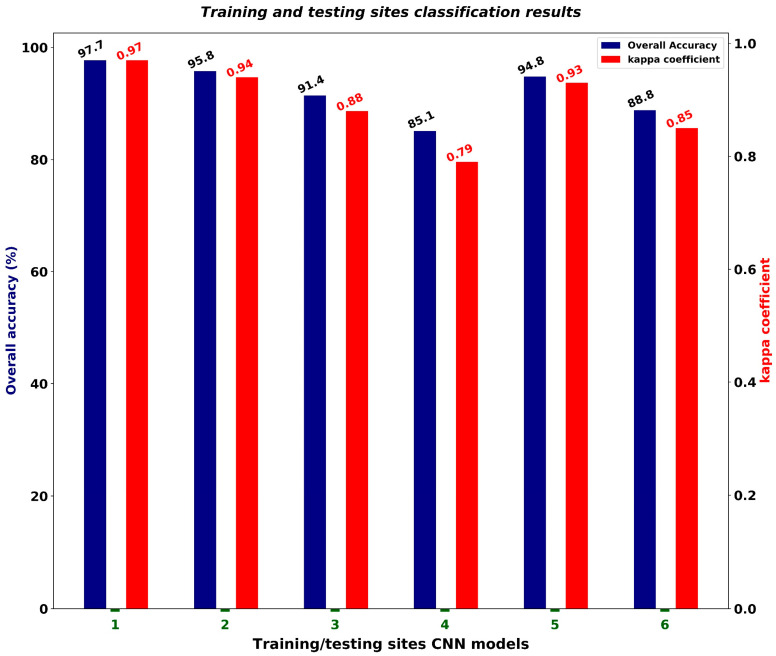
Training and testing site classification results of 4–10-band composite CNN models. On the x-axis, numerical values represent the following. 1: Training site (4-band composite CNN model); 2: Training site (10-band composite CNN model); 3: Testing site 1: Faisalabad city (4-band composite CNN model); 4: Testing site 1: Faisalabad city (10-band composite CNN model); 5: Testing site 2: Lahore city (4-band composite CNN model); 6: Testing site 2: Lahore city (10-band composite CNN model).

The highest OA, 97.7%, is achieved by the 4-band CNN model (kappa coefficient = 0.97) (Table 4). For the 4-band CNN model, the barren land class has the lowest PA with 94.3%. It misclassified 1.7% of pixels as fallow land class and 4% as settlement class (Table 5). These are highly spectrally confused LULC classes. The settlement, vegetation, and water bodies classes have the highest PA accuracy among all the LULC classes; it is difficult to classify settlement class. Our proposed CNN model accurately classified the unplanned settlement class in this region. For the 10-band CNN model, barren land has the lowest PA with 87.3%. It misclassified 5.5% of pixels as fallow land class and 6.3% as settlement (Table 6). These results show that the 4-band CNN model is better than the 10-band CNN model in terms of classification accuracy. The 4-band CNN model has classified spectrally confused LULC classes with higher accuracy. The 4-band CNN model had a computational time of 2 min 17 s, which is lower than the 10-band CNN model’s computational time (3 min 42 s) (Table 4). The graphical representation of results (Table 4) is shown in Figure A1 (Appendix A).

### 3.3. The Trained 4–10-Band CNN Models’ Prediction on Unseen Sites

**Figure 7:**
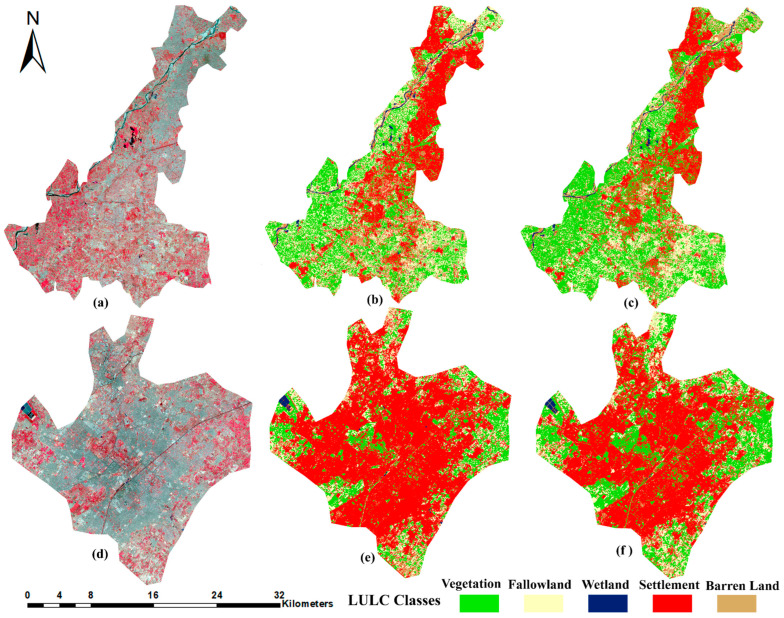
LULC maps of trained 4- and 10-band CNN model, applied to two test sites. (**a**,**d**) show the false-color composite images of testing sites, Lahore and Faisalabad city, respectively. (**b**,**c**,**e**,**f**) show the classification results of 4- and 10-band CNN model for Lahore and Faisalabad city, respectively.

**Figure 8:**
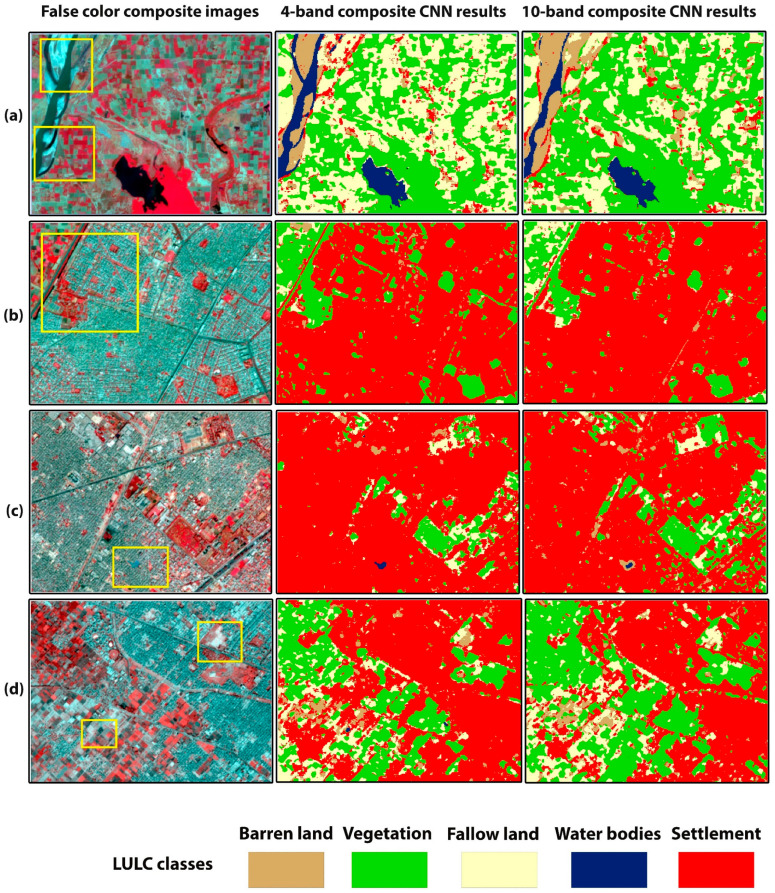
Subsets of the LULC maps of the test sites. First column shows the false-color composite images. (**a**,**b**) are the classification maps of Lahore city, (**c**,**d**) maps of Faisalabad city. The yellow rectangles highlight areas with differences between the classification results of 4- and 10- band CNN models.

We have tested the trained 4- and 10-band CNN models on two cities: Lahore and Faisalabad. Figure 7 shows the comparison between the 4- and 10-band CNN models’ classification results of Lahore and Faisalabad. Figure 8 shows smaller subsets of testing site classification results in more detail. Figure 8a,b depicts the LULC maps of Lahore city. As shown in Figure 8a, the area mainly comprises river, vegetation, and fallow land features. In the rectangle’s area, the 10-band CNN model shows compactness in the river area and misclassifies the river pixels as settlements. The 4-band CNN correctly classified these land covers. In Figure 8b, the 4-band CNN classified the road as settlement, but the 10-band CNN classified the road pixels as vegetation pixels. Figure 8c,d shows the LULC map of Faisalabad city. Figure 8c,d represents the urban, vegetation, and fallow land areas. The 10-band CNN model misclassified the water bodies area as barren land, and the barren land area as fallow land. The 4-band CNN model correctly classified these classes more than the 10-band CNN model. The highest OA, 94.8% and 91.4%, was achieved by the 4-band CNN model for Lahore and Faisalabad city images, respectively (Table 7). For Lahore city, the highest PA was achieved by the settlement class with 99.1%, and the lowest PA, 90.3% and 90.4%, was achieved by the fallow land and barren land classes respectively. For barren land, 7.8% and 1.7% of pixels were misclassified as settlement and fallow land classes, respectively. For fallow land, 6.89%, 1.37%, and 1.37% of pixels were misclassified as vegetation, barren land, and settlement class, respectively (Table 8). The 10-band CNN model has achieved the lowest PA, with 67.2% and 83.4% for barren land and water bodies classes, respectively. In the case of barren land, 14.1%, 15.9% and 2.65% of pixels were misclassified as settlement, fallow land, and vegetation class pixels respectively. For water bodies, 11.7%, 2.9% and 1.9% of pixels were misclassified as barren land, settlement, and fallow land classes, respectively (Table 9). The 4-band CNN model prediction results are better in terms of classification accuracy than the 10-band CNN model results. For Faisalabad city, the highest and lowest PA was achieved by the settlement class, with 99%, and barren land class, with 61.3%. For barren land, 21.3% and 17.4% of pixels were misclassified as settlement and fallow land class pixels, respectively (Table 10). The 10-band CNN achieved the lowest PA of 46.37% for the barren land class and 68.5% for the water bodies class. For barren land, 20.2% and 33.43% of pixels were misclassified as settlement and fallow land classes, respectively. For water bodies, 25.71%, 3.8%, and 1.99% of pixels were misclassified as barren land, settlement, and vegetation classes, respectively (Table 11). The 4- and 10-band CNN model prediction results on unseen data demonstrate that the 4-band CNN model prediction results are better than the 10-band CNN model prediction results in terms of classification accuracy. The misclassification between the spectrally confused LULC classes is higher in 10-band CNN model results than in the 4-band CNN model results. The graphical representation of results (Table 7) is shown in Figure A1.

## 4. Discussion

The aim of this study was to analyze the spectral bands of Sentinel-2 imagery for semi-arid region classification problems, e.g., which spectral band combination can reduce the spectral confusion between spectrally confused LULC classes. A 2D-patch-based CNN with fixed architecture and fine-tuned hyperparameters was used for classification. In total, 2400 training patches of 5 × 5 pixel size were manually labeled for each LULC class (Table 2). The 4–10-band composite CNN models were trained. It has been observed that 4-band CNN performed better in terms of classification accuracy than the 10-band CNN model. The 10-band CNN model has produced acceptable results. As shown in Figure 5 and Figure 6, there is more misclassification between spectrally confused LULC classes (barren land, settlement, and fallow land) in 10-band CNN model results as compared to the 4-band CNN model results. An OA of 97.7% and 95.8% is achieved by the 4- and 10-band CNN models, respectively (Table 4). Table 5 and Table 6 describe the per class PA and UA of every LULC class for 4–10-band CNN models. We have achieved the highest PA for settlement (99.4%), fallow land (96%), and barren land (94.3%) classes, as the classified results can be seen in Figure 5 and Figure 6. These are the highly spectrally confused LULC classes.

We have tested the trained 4–10-band models on two out-of-sample semi-arid cities: Lahore and Faisalabad. Figure 7 and Figure 8 show the comparison between the 4- and 10-band CNN model classification results of Lahore and Faisalabad. A highest OA of 94.8% and 91.4% was achieved by the 4-band CNN model for Lahore and Faisalabad city images, respectively (Table 7). Per class UA and PA of 4- and 10-band CNN models for Lahore and Faisalabad city images are described in Table 8, Table 9, Table 10 and Table 11. We have achieved promising results on testing sites. This is evidence of our right approach to the preparation of the dataset and the proposed CNN model with fixed architecture and tuned hyperparameters.

The reason for the 10-band CNN model classification accuracy being lower than the 4-band CNN model is due to the lower spatial resolution of the additional six 20 m bands, which were down-sampled to 10 m spatial resolution in this study using the nearest neighbor resampling method. The nearest neighbor is an efficient resampling method for downscaling of spectral bands [47,48], and it has the advantage of preserving and making few alternations to the original pixel values of resampled bands as compared to the other resampling methods [49]. However, this down-sampling process does not increase the resolution of the original image bands, it merely modifies them to have a smaller pixel size. Band resampling is a very time-consuming process and requires computational power, especially for large-area images. Applying more complicated image fusion algorithms—e.g., image pan sharpening of the 20 m bands—may lead to better classification performance, but requires even more computational power and sometimes leads to spectral distortion of the down-sampled image bands [39,50]. That said, future studies could compare the accuracy of 4-band and 10-band Sentinel-2 composite images after applying more complex image fusion algorithms to better understand the utility of the additional six lower-resolution image bands for LULC mapping in semi-arid regions.

## 5. Conclusions

It is very difficult to separate spectrally confused land-cover classes in semi-arid regions using medium-resolution remotely sensed data, as the spectral response of several classes (e.g., settlements, barren land, and fallow land) are highly similar. In this study, we used a CNN model with fixed architecture to perform LULC classification in three study sites in Pakistan. The first study site (consisting of Gujranwala city and Gujranwala Saddar and Wazirabadtownships, Pakistan) was used as a training site for tuning the CNN’s hyperparameters, and the optimized CNN was then applied to two unseen testing sites (Lahore city and Faisalabad city, Pakistan) to evaluate the robustness of our proposed classification approach in semi-arid regions with complex LULC compositions. This study also evaluated the efficacy of different band combinations of Sentinel-2 imagery for LULC classification in these semi-arid regions. In training sites, our experimental results showed that a 4-band CNN model (blue, green, red, and near-infrared bands) with the proposed CNN architecture achieved an overall classification accuracy of 97.7% (kappa coefficient = 0.97), outperforming the 10-band CNN model (overall accuracy = 95.8%, kappa coefficient = 0.94). In the two testing sites, the trained 4- and 10-band CNN models achieved overall classification accuracies of 94.8% and 88.8%, respectively, for Lahore city, and 91.4% and 85.1%, respectively, for Faisalabad city. The results showed that the 4-band (10 m spatial resolution) CNN model was more suitable for separating the spectrally confusing LULC classes in the training and testing sites, as it achieved higher classification accuracy and required lower computational power and training time than the 10-band CNN model. Although we have only focused on the use of CNNs for semi-arid LULC mapping in this study, other Machine Learning and Deep Learning methods may also be able to achieve similar or better performance under different circumstances (e.g., considering different training data sizes/different types of LULC classes). Future works could focus on comparing our optimal 4-band CNN model with other classification models to better understand these factors. It is hoped that our findings can be helpful for future studies involving the mapping of LULC in semi-arid regions using Sentinel-2 or other medium-resolution satellite imagery.

## Figures and Tables

**Table 1:** Details of Sentinel-2 bands used in this research.

Spectral Bands	Central Wavelength (nm)	Spatial Resolution (m)
Band 2: Blue	0.409	10
Band 3: Green	0.56	10
Band 4: Red	0.665	10
Band 5: Vegetation Red-Edge	0.705	20
Band 6: Vegetation Red-Edge	0.74	20
Band 7: Vegetation Red-Edge	0.783	20
Band 8: Near infrared	0.842	10
Band 8A: Vegetation Red-Edge	0.865	20
Band 11: SWIR	1.61	20
Band 12: SWIR	2.19	20

**Table 2:** Details of training samples used for LULC classification.

LULC Classes	Training Patches (5 × 5) Pixels
Settlement	2400
Barren land	2400
Fallow land	2400
Vegetation	2400
Water bodies	2400

**Table 3:** Simulation parameters.

Parameter	Value
Dropout	0.2, 0.5
Learning Rate	0.0001
Epochs	300
Batch Size	128
Activation Functions	ReLu, softmax
Loss Function	categorical cross entropy
Optimizer	Adam

**Table 4:** OA (%), kappa measures, and training time of 4–10-band CNN models.

Model	OA	Kappa Coefficient	Training Time
4-band CNN	97.7	0.97	2 min 17 s
10-band CNN	95.8	0.94	3 min 42 s

**Table 5:** The confusion matrix of the training site classification results obtained by 4-band CNN model.

LULC Classes	Barren Land	Settlement	Fallow Land	Vegetation	Water Bodies	Sum	UA (%)
Barren land	116	1	8	0	0	125	92.8
Settlement	5	193	0	0	2	200	96.5
Fallow land	2	0	197	1	0	200	98.5
Vegetation	0	0	0	200	0	200	100
Water bodies	0	0	0	0	125	125	100
Sum	123	194	205	201	127	850	
PA (%)	94.3	99.4	96	99.5	98.4		

**Table 6:** The confusion matrix of the training sites classification results obtained by 10-band CNN model.

LULC Classes	Barren Land	Settlement	Fallow Land	Vegetation	Water Bodies	Sum	UA (%)
Barren land	110	2	1	0	4	117	94
Settlement	8	186	1	0	4	199	93.46
Fallow land	7	2	202	1	0	212	95.28
Vegetation	1	1	3	197	0	202	97.5
Water bodies	0	0	0	0	120	120	100
Sum	126	191	207	198	128	850	
PA (%)	87.3	97.3	97.58	99.4	93.75		

**Table 7:** OA (%), kappa coefficient of 4–10-band CNN models for the Lahore and Faisalabad city images.

Testing Sites	Model	OA (%)	Kappa Coefficient
Lahore city	4-band CNN	94.8	0.93
	10-band CNN	88.8	0.85
Faisalabad city	4-band CNN	91.4	0.88
	10-band CNN	85.1	0.79

**Table 8:** The confusion matrix of the testing site (Lahore city) classification results obtained by 4-band CNN model.

LULC Classes	Barren Land	Settlement	Fallow Land	Vegetation	Water Bodies	Sum	UA (%)
Barren land	104	0	2	0	0	106	98.1
Settlement	9	225	2	0	1	237	94.9
Fallow land	2	2	131	13	3	151	86.75
Vegetation	0	0	10	247	0	257	96.1
Water bodies	0	0	0	0	99	99	100
Sum	115	227	145	260	103	850	
PA (%)	90.4	99.1	90.3	95	96.11		

**Table 9:** The confusion matrix of the testing site (Lahore city) classification results obtained by 10-band CNN model.

LULC Classes	Barren Land	Settlement	Fallow Land	Vegetation	Water Bodies	Sum	UA (%)
Barren land	76	1	8	0	12	97	78.3
Settlement	16	207	0	0	3	226	91.5
Fallow land	18	12	132	7	2	171	77.1
Vegetation	3	4	9	254	0	270	94
Water bodies	0	0	0	0	86	86	100
Sum	113	224	149	261	103	850	
PA (%)	67.2	92.4	88.5	97.3	83.4		

**Table 10:** The confusion matrix of the testing site (Faisalabad city) classification results obtained by 4-band CNN model.

LULC Classes	Barren Land	Settlement	Fallow Land	Vegetation	Water Bodies	Sum	UA (%)
Barren land	46	0	0	2	0	48	95.8
Settlement	16	319	0	0	0	335	95.2
Fallow land	13	3	89	34	2	141	63.1
Vegetation	0	0	3	213	0	216	98.6
Water bodies	0	0	0	0	110	110	100
Sum	75	322	92	249	112	850	
PA (%)	61.3	99	96.7	85.5	98.2		

**Table 11:** The confusion matrix of the testing site (Faisalabad city) classification results obtained by 10-band CNN model.

LULC Classes	Barren Land	Settlement	Fallow Land	Vegetation	Water Bodies	Sum	UA (%)
Barren land	32	2	2	0	27	63	50.7
Settlement	14	306	0	1	4	325	94.1
Fallow land	23	7	78	30	0	138	56.52
Vegetation	0	4	10	236	2	252	93.65
Water bodies	0	0	0	0	72	72	100
Sum	69	319	90	267	105	850	
PA (%)	46.37	95.9	86.6	88.38	68.5		

## Data Availability

The satellite images used in this study are freely available from the European Space Agency’s (ESA) website (https://scihub.copernicus.eu/), and the LULC maps of study sites are available upon request.
